# Is there any association between gut microbiota and type 1 diabetes? A systematic review

**DOI:** 10.1186/s13099-019-0332-7

**Published:** 2019-10-14

**Authors:** Parnian Jamshidi, Saba Hasanzadeh, Azin Tahvildari, Yeganeh Farsi, Mahta Arbabi, João Felipe Mota, Leonardo A. Sechi, Mohammad Javad Nasiri

**Affiliations:** 1 Student Research Committee, School of Medicine, Shahid Beheshti University of Medical Sciences, Tehran, Iran; 2 Clinical and Sports Nutrition Research Laboratory (LABINCE), Faculty of Nutrition, Federal University of Goiás, Goiânia, Brazil; 3 Department of Biomedical Sciences, University of Sassari, Sassari, Italy; 4 Department of Microbiology, School of Medicine, Shahid Beheshti University of Medical Sciences, Tehran, Iran

**Keywords:** Type 1 diabetes, Microbiota, Dysbiosis

## Abstract

**Introduction:**

Type 1 diabetes (T1D) is the second most common autoimmune disease among children. There is evidence suggesting that dysbiosis of some gut colonizing bacteria are associated with the pathogenesis of T1D. However, these studies are still controversial and a systematic review was conducted to evaluate the association between gut microbiota and T1D.

**Methods:**

A systematic search was carried out in Medline (Via Pubmed) and Embase from January 2000 to January 2019 for all original cross-sectional, cohort, case–control or nested case–control studies investigating the association between gut microbiota and T1D.

**Results:**

Of 568 articles identified, 26 studies met the inclusion criteria. The total population study of these articles consists of 2600 children (under 18 years old) and 189 adults. Among the included studies, 24 articles confirmed the association between gut microbiota dysbiosis and T1D. The most common bacterial alterations in T1D patients included *Bacteroides* spp., *Streptococcus* spp., *Clostridium* spp., *Bifidobacterium* spp., *Prevotella* spp., *Staphylococcus* spp., *Blautia* spp., *Faecalibacterium* spp., *Roseburia* spp., and *Lactobacillus* spp.

**Conclusion:**

Our study showed a significant association between alterations in intestinal microbial composition and T1D; however, in some articles, it is not clear which one happens first. Investigation of altered gut microbiota can help in the early detection of T1D before seropositivity. Targeted microbiome modulation can be a novel potential therapeutic strategy.

## Introduction

Type 1 diabetes (T1D) is the second most common autoimmune disease among children. It is accompanied by many complications and has life-long morbidity [1]. The incidence of T1D is increasing universally and accounts for 5–10% of all diabetic morbidity [2]. T1D is a chronic autoimmune inflammatory process that affects insulin-producing beta cells of the pancreas, results in less insulin production [3]. Destruction of 90% of beta cells is a critical point that clinical manifestations emerge [4]. Because of the early onset of disease and chronicity, T1D is of great importance. Previous animal and human studies have shown the role of genetic factors like human leukocyte antigen (HLA) DQ and DRB in the pathogenesis of disease but recent studies propose the significant role of environmental factors such as gut colonizing bacteria [5]. Gut microbiota has an important role in the regulation of metabolism, systemic and local immunity [6]. From birth to age 3, gut microbiota undergoes a lot of changes and the microbiota composition of a 3-year-old child is similar to that of an adult [7]. The most important factors affecting gut microbiota include the type of delivery [8], breastfeeding [9] or bottle feeding, maternal microbiota composition, mother’s diet during pregnancy and the western diet [5, 10–13], contact with peers, environment, and use of antibiotics [14–17]. Gut dysbiosis, an imbalance of the microbial communities, can be associated with metabolic disorders, obesity, insulin resistance, Type 2 diabetes (T2D), inflammatory bowel disease, celiac disease and immunity dysfunction [18–20]. Lately, there is evidence suggesting the correlation between dysbiosis and pathogenesis of T1D [21]. However, these studies are still controversial and need further investigation; Thus, we carried out a systematic review about the association between gut microbiota and T1D according to the Preferred Reporting Items for Systematic Reviews and Meta-Analyses statement [22].

## Materials and methods

### Search strategy

A systematic search was carried out in Medline (Via Pubmed) and Embase from January 2000 to January 2019. Medical Subject Headings (MeSH) were “gastrointestinal microbes”, “dysbiosis”, “gut microbiota”, “gut bacteria”, “gut microbes” combined with “type 1 diabetes mellitus”. Lists of references of selected articles and relevant review articles were hand-searched to identify further studies. Only studies written in English were selected.

### Study selection

Two reviewers independently performed the review of titles and abstracts and chose those fitting selection criteria for full-text evaluation. Discrepancies were discussed with a third reviewer. All original cross-sectional, cohort, case–control or nested case–control studies investigating the association between gut microbiota and T1D patients were considered. The following articles were excluded: animal studies, case reports, reviews, and editorials.

### Data extraction

The following variables were extracted: first author; year of publication; study duration, type of study, country/ies where the study was conducted; the number of cases with T1D; age; gender; microbiota analysis technique; modifications of intestinal microbiota and modifications of biochemical and immunological factors. Data were independently collected by two authors.

## Results

**Fig. 1:**
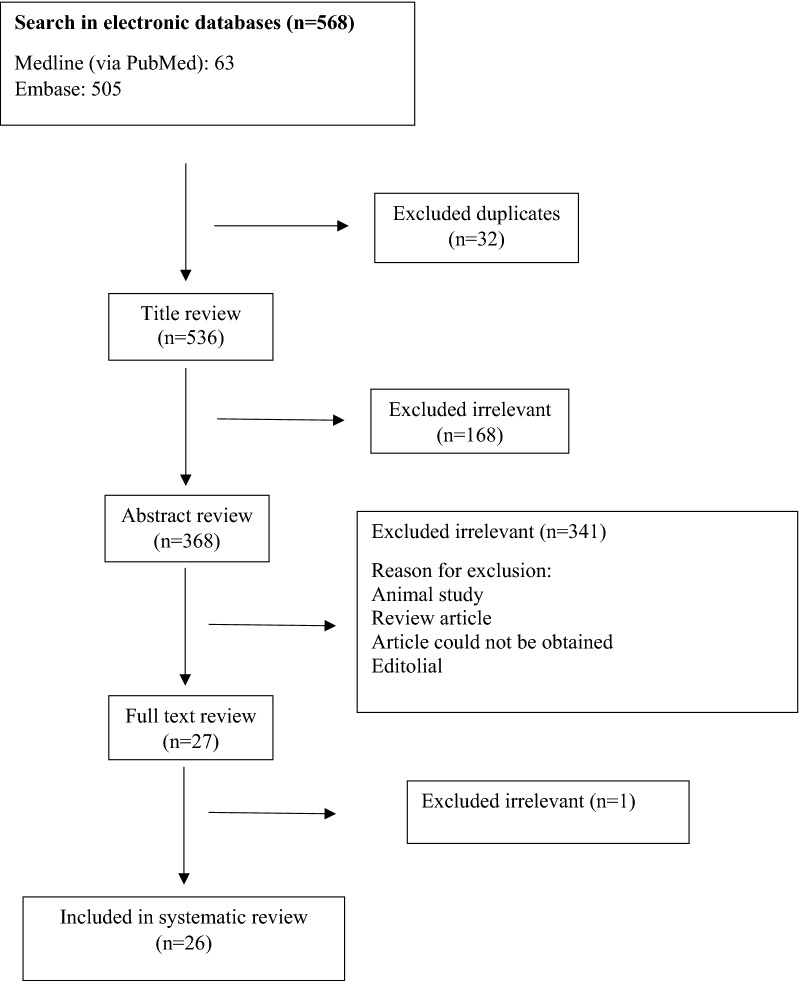
Flow chart of study selection for inclusion in the systematic review

The selection process of articles is shown in Fig. 1. Twenty-six articles were included and classified into 16 case–control studies [18, 21, 23–36], 6 cohort studies [37–42], 2 cross-sectional studies [5, 43] and 2 nested case–control studies [44, 45]. Four of these studies were conducted in the USA, three in Italy, three in Finland, two in China, two in Spain and others in Netherland, Germany, Turkey, UK, Portugal, Poland, Russia, Mexico, Brazil, Australia, Czech Federation, and France. The population of these articles consists of 2600 children (under 18 years old) and 189 adults. The most applied techniques for detection and assessment of gut microbiota in stool samples where PCR, real-time quantitative PCR, 16s rRNA sequencing, microarray analysis, proteomics and quantitative cultures of stool samples (Table 1).

**Table 1:** Characteristics of included studies

Authors	Year	Country	Type of study	Study population (control and case)	Age (mean)	Microbiota analysis technique
Rozanova et al. [41]	2002	Russia	Cohort	38 T1D	3 years	Not mentioned
Brown et al. [23]	2011	Finland	Case–control	8 T1D	Children	DNA sequencing
Giongo et al. [31]	2011	USA	Case–control	Control: 4, case: 4	5 months	16s rRNA sequencing
Murri et al. [26]	2013	Spain	Case–control	Control: 16, case: 16	7 years	Real time quantitative PCR
Richardson et al. [37]	2014	Finland	Cohort	Control: 47, case: 29	2 years	16s rRNA sequencing
de Goffau et al. [24]	2014	Netherlands	Case–control	Control: 27, case: 28	3 years	Microarray analysis
Endesfelder et al. [38]	2014	Germany	Cohort	Control: 22, case: 22	19.5 months	16s rRNA sequencing
Mejia et al. [30]	2014	Mexico	Case–control	Control: 8, case: 21	12.5 years	16s rRNA pyrosequencing
Soyucen et al. [28]	2014	Turkey	Case–control	Control: 35, case: 35	10 years	Quantitative cultures on selective and non-selective media
Kostic et al. [39]	2015	Finland	Cohort	Control: 22, case: 11^b^	Infants	16s rRNA sequencing
Alkanani et al. [5]	2015	USA	Cross-sectional	Control: 23, case^a^: 88	11 years	16s rRNA sequencing
Cui et al. [18]	2016	China	Case–control	Control: 15, case: 15	11 years	16s rRNA sequencing
Maffeis et al. [25]	2016	Italy	Case–control	Control: 10, case: 10	11 years	Semi-quantitative PCR
Stewart et al. [29]	2017	UK	Case–control	Control: 10, case: 10	27 years	16s rRNA sequencing
Pinto et al. [40]	2017	Portugal	Cohort	Control: 3, case: 3	9 years	Real time quantitative PCR
Pellegrini et al. [27]	2017	Italy	Case–control	Control: 35^c^, case: 19	36 years	Real time quantitative PCR
Traversi et al. [21]	2017	Italy	Case–control	Control: 13, case: 13	8 years	Real time quantitative PCR
Gao et al. [42]	2018	France	Cohort	33 genetically predisposed to T1D	1.5 years	16s rRNA sequencing
Vatanen et al. [44]	2018	USA	Nested case–control	Control: 415, case: 368^d^	3 month	16s rRNA sequencing
Stewart et al. [45]	2018	USA	Nested case–control	903 children	24.5 month	16s rRNA sequencing
Huang et al. [32]	2018	China	Case–control	Control: 10, case: 12	23.5	16s rRNA sequencing
Gavin et al. [43]	2018	Australia	Cross-sectional	Control: 22, case: 69^e^	10.8	Proteomics and 16S rRNA sequencing
Leiva-Gea et al. [33]	2018	Spain	Case–control	Control: 13, case: 15 T1D	12.6	16S rRNA pyrosequencing
Higuchi et al. [34]	2018	Brazil	Case–control	Control: 28, case: 20	23.1	16s rRNA sequencing
Salamon et al. [35]	2018	Poland	Case–control	Control: 23, case: 22	42.5	16s rRNA sequencing
Cinek et al. [36]	2018	Czech federation	Case–control	Control: 103, case: 73^f^	11.8	16s rRNA sequencing

^a^35 new-onset patients; 21 seropositive; 32 seronegative FDRs (first degree relatives)

^b^Seroconverters

^c^16 healthy control; 19 gut inflammatory disease as the second control

^d^267 seroconverters and 101 diagnosed with T1D

^e^23 recent onset type 1 diabetes; 17 islet autoantibody–positive subjects; 29 low-risk autoantibody-negative subjects

^f^Azerbaijan: 19, Jordan: 20, Nigeria: 14, Sudan: 20

### Gut microbiota and type 1 diabetes

Twenty-four out of twenty-six articles confirmed the association of T1D and gut microbiota dysbiosis. In one study alterations could not be attributable to T1D [23] and one of the articles is only a preliminary study and doesn’t have any obvious conclusion yet [21] (Table 2). The most common bacterial alterations in T1D patients group versus healthy individuals included *Bacteroides* spp., *Streptococcus* spp., *Clostridium* spp., *Bifidobacterium* spp., *Prevotella* spp., *Staphylococcus* spp., *Blautia* spp., *Faecalibacterium* spp., *Roseburia* spp., and *Lactobacillus* spp. Details indicating the altered bacteria are shown in Table 3.

**Table 2:** Association of gut microbiota and type 1 diabetes

Authors	Year	Country	Type of study	Association between diabetes and microbiome
Rozanova et al. [41]	2002	Russia	Cohort	Yes
Brown et al. [23]	2011	Finland	Case–control	May
Giongo et al. [31]	2011	USA	Case–control	Yes
Murri et al. [26]	2013	Spain	Case–control	Yes
Richardson et al. [37]	2014	Finland	Cohort	Yes
de Goffau et al. [24]	2014	Netherlands	Case–control	Yes
Endesfelder et al. [38]	2014	Germany	Cohort	Yes
Mejia et al. [30]	2014	Mexico	Case–control	Yes
Soyucen et al. [28]	2014	Turkey	Case–control	Yes
Kostic et al. [39]	2015	Finland	Cohort	Yes
Alkanani et al. [5]	2015	USA	Cross-sectional	Yes
Cui et al. [18]	2016	China	Case–control	Yes
Maffeis et al. [25]	2016	Italy	Case–control	Yes
Stewart et al. [29]	2017	UK	Case–control	Yes
Pinto et al. [40]	2017	Portugal	Cohort	Yes
Pellegrini et al. [27]	2017	Italy	Case–control	Yes
Traversi et al. [21]	2017	Italy	Case–control	Not mentioned
Gao et al. [42]	2018	France	Cohort	Yes
Vatanen et al. [44]	2018	USA	Nested case–control	Yes
Stewart et al. [45]	2018	USA	Nested case–control	Yes
Huang et al. [32]	2018	China	Case–control	Yes
Gavin et al. [43]	2018	Australia	Cross-sectional	Yes
Leiva-Gea et al. [33]	2018	Spain	Case–control	Yes
Higuchi et al. [34]	2018	Brazil	Case–control	Yes
Salamon et al. [35]	2018	Poland	Case–control	Yes
Cinek et al. [36]	2018	Czech federation	Case–control	Yes

**Table 3:** Intestinal microbiota modifications

Authors	Intestinal microbiota modifications
Rozanova et al. [41]	
Increased	Lactose-negative Enterobacteriaceae, *Klebsiella* spp., *Enterococcus* spp., *Candida* spp., *Clostridium* spp., *Staphylococcus epidermidis*
Decreased	*Bifidobacterium* spp., *Lactobacillus* spp., *Escherichia coli*
Brown et al. [23]	
Increased	*Bacteroides* spp., *Veillonella* spp., *Alistipes* spp.
Decreased	*Prevotella* spp., *Akkermansia* spp.
Giongo et al. [31]	
Increased	Bacteroidetes
Decreased	Firmicutes
Murri et al. [26]	
Increased	*Clostridium* spp., *Bacteroides* spp. and *Veillonella* spp.
Decreased	*Lactobacillus* spp., *Bifidobacterium* spp., *Blautia coccoides*, *Eubacterium rectale*, *Prevotella* spp., lactic acid-producing bacteria, butyrate-producing bacteria and mucin-degrading bacteria
Richardson et al. [37]	
Increased	*Bacteroides dorei*, *Bacteroides vulgatus*
Decreased	–
de Goffau et al. [24]^a^	
Increased	*Streptococcus mitis*, Bacteroidetes
Decreased	–
de Goffau et al. [24]^b^	
Increased	Non butyrate producing species of Clostridium cluster *14a*, *Clostridium stercorarium*
Decreased	–
Endesfelder et al. [38]^c^	
Increased	*Enterococcus* spp., *Sarcina* spp., *Prevotella* spp., *Corynebacterium* spp.
Decreased	–
Endesfelder et al. [38]^d^	
Increased	*Barnesiella* spp., *Candidatus Nardonella*
Decreased	*Staphylococcus* spp., *Nocardioides* spp.
Mejia et al. [30]	
Increased	*Bacteroides* spp.
Decreased	*Prevotella* spp.
Soyucen et al. [28]	
Increased	Enterobacteriaceae, *Candida albicans*
Decreased	*Bifidobacterium* spp.
Kostic et al. [39]	
Increased	–
Decreased	*Coprococcus eutactus*, *Dialister invisus*
Alkanani et al. [5]	
Increased	*Lactobacillus* spp., *Staphylococcus* spp.
Decreased	Prevotellaceae
Cui et al. [18]	
Increased	*Blautia* spp., *Haemophilus* spp., *Lachnospira* spp., *Intestinimonas* spp., *Dialister* spp., *Micrococcales* spp.
Decreased	*Pasteurella* spp., *Caulobacterales* spp.
Maffeis et al. [25]	
Increased	*Dialister invisus*, *Globicatella sanguinis*, *Bifidobacterium longum*
Decreased	–
Stewart et al. [29]	
Increased	*Actinomyces* spp.
Decreased	–
Pinto et al. [40]	
Increased	*Eubacterium rectale*, *Faecalibacterium prausnitzzi*, *Bacteroides dorei*, *Bacteroides uniformis*
Decreased	*Collinsella aerofaciens*, *Coprococcus Comes*, *Clostridium* spp., *Bifidobacterium adolescentis*, *Bifidobacterium longum* *infantis*, *Ruminococcus* spp., *Collinsella* spp.
Pellegrini et al. [27]	
Increased	Firmicutes
Decreased	*Clostridium* spp., Bacteroidetes, Proteobacteria
Traversi et al. [21]	
Increased	*Bacteroides clarus*, *Alistipes obesi*, *Bifidobacterium longus*, *Methanobrevibacter Smithii*
Decreased	*Bacteroides coprophilus*, *Bacteroides dorei*, *Fusicatenibacter saccharivorans*, *Bacteroides vulgatus*, *Bacteroides oleiciplenus*, Firmicutes
Gao et al. [42]	This study emphasizes on interactions between gut microbiota rather than quantitative changes
Vatanen et al. [44]	
Increased	*Bifidobacterium pseudocatenulatum*, *Roseburia hominis*, *Alistipes shahii*
Decreased	*Streptococcus thermophilus*, *Lactococcus lactis*
Stewart et al. [45]	
Increased	–
Decreased	*Ruminococcus* spp., *Lactococcus* spp., *Streptococcus* spp., *Akkermansia* spp.
Huang et al. [32]	
Increased	Bacteroidetes/Firmicutes ratio, Porphyromonadaceae
Decreased	*Ruminococcus* spp., *Veillonella* spp., *Phascolarctobacterium* spp., *Fusobacterium* spp., Paenibacillaceae
Gavin et al. [43]	
Increased	*Bacteroides* spp., *Prevotella* spp.
Decreased	*Alistipes* spp., *Ruminococcus* spp., *Barnesiella* spp., *Clostridium* spp., *Dorea* spp., *Faecalibacterium Prausnitzii*
Leiva-Gea et al. [33]	
Increased	*Bacteroides* spp., Rikenellaceae, *Ruminococcus* spp., *Veillonella* spp., Enterobacteriaceae, *Blautia* spp., *Streptococcus* spp., Prevotellaceae, *Sutterella* spp.
Decreased	*Bifidobacterium* spp., *Roseburia* spp., *Faecalibacterium* spp., *Lachnospira* spp., *Anaerostipes* spp., Actinobacteria, Proteobacteria, Firmicutes
Higuchi et al. [34]	
Increased	*Bacteroides* spp., *Alistipes* spp., *Prevotella* spp.
Decreased	–
Salamon et al. [35]	
Increased	*Akkermansia* spp., *Ruminococcus* spp., *Bacteroides* spp., *Blautia* spp.
Decreased	*Lachnospira* spp., *Faecalibacterium* spp., *Bifidobacterium* spp., *Coprococcus* spp., *Collinsella* spp., *Dorea* spp.
Cinek et al. [36]	
Increased	*Escherichia coli*
Decreased	*Eubacterium* spp., *Roseburia* spp., *Haemophilus* spp., Clostridium clusters IV and XIVa

^a^In cases with < 2.9 years

^b^In cases with > 2.9 years

^c^Eigenvector centrality (EC) at age 0.5 years

^d^EC at age 2 years

### The relationship between intestinal microbiota and HbA1C, inflammatory mediators and serum zonulin level

Some articles reported evidence of an association between HbA1C level and bacterial groups such as *Blautia* spp. count and Firmicutes:Bacteroidetes ratio (F:B ratio) [18, 33]. Murri et al. [26] in 2013 by designing a case–control study noticed the HbA1C level affected *Clostridium* spp. positively and F:B ratio negatively in both uni and multivariant statistical analysis. Univariate statistical analysis also showed that *Bifidobacterium* spp. and *Lactobacillus* spp. may affect HbA1C levels [26]. On the contrary, Alkanani et al. [5] reported that bacterial alterations in the case group were not associated with the HbA1C level.

There is an elevated level of TNF-α expression in lamina propria of intestinal biopsy in T1D patients in comparison with healthy individuals [27]. Higuchi et al. [34] reported a negative correlation between TNF plasma level and Proteobacteria and Clostridiaceae abundance. In another study, increment of *Bacteroides* spp. and decrement of *Roseburia* spp. was correlated with TNF-α level [33].

Interleukin-6 has an important correlation with Ruminococcaceae abundance as reported by the Higuchi et al. study [34]. Increase of *Bacteroides* spp. and decrease of *Roseburia* spp. abundance is correlated with serum IL-6 level [33].

According to Leiva-Gea et al. [33] an increase in *Bacteroides* spp. and *Veillonella* spp. and decrease in *Bifidobacterium* spp., *Roseburia* spp. and *Faecalibacterium* spp. was associated with serum IL-1β; in addition, an increase of *Streptococcus* spp. and decrease of *Bifidobacterium* spp. was reported related to serum IL-10 and IL-13 levels.

Serum zonulin level has a significant role in the pathogenesis of T1D. Leiva-Gea et al. [33] showed that an increase in *Bacteroides* spp. and *Veillonella* spp. and decrease in *Faecalibacterium* spp. and *Roseburia* spp. was correlated with an increased serum zonulin level.

## Discussion

We reviewed 26 articles, twenty-four of them approved a straight correlation between microbiota and diabetes; however, most of them didn’t clarify if microbiota induces T1D or T1D changes gut microbiome. The articles were screened according to the type of gut microbiota and correlation with T1D as explained below: one article mentioned that microbiome alteration occurs after diabetes [26], two articles studied microbiota as a therapeutic agent on T1D [35, 41], seven articles just showed the differences in gut microbiota of healthy and diabetic people and didn’t discuss the type of relation [21, 24, 29, 32, 34, 36, 40], finally fourteen articles suggested the exact mechanism that leads to autoimmunity by the change in gut microbiome [5, 18, 25, 27, 28, 31, 33, 37, 39, 42–46] (one article was just in the abstract form and we couldn’t read the details [30]).

Different mechanisms have been suggested about the role of gut microbiota in the pathogenesis of T1D. These mechanisms are mainly derived from the 14 articles mentioned above. In more details, the following points can be noticed:

1. In patients with T1D, some bacteria increase mucin degradation, results in reduced integrity and increased permeability of intestinal mucosa that leads to bacterial penetration [47]. The penetration of bacteria into intestinal mucosa leads to stimulation of the immune system and production of antibodies against them [47]. Cross-reaction of these antibodies and surface antigens of pancreatic beta cells, as well as T cell cross-reactivity results in the destruction of beta cells and formation of T1D [47].
2. Butyrate is one of the most important byproducts of microbiota metabolisms and plays an important role in colonic T-reg induction, down-regulation of pro-inflammatory macrophages and integrity enhancement of gut barriers through increasing mucin production [48, 49].
3. Zonulin is a protein that can be assumed as an important indicator of mucosal integrity and gut permeability [33]. This protein modulates intercellular junctions and macromolecular passage through them [33]. Some bacterial groups can alter mucosal integrity by affecting zonulin; increase in *Bacteroides* spp. and *Veillonella* spp. or decrease in *Faecalibacterium* spp. and *Roseburia* spp. correlates with increased serum zonulin levels in T1D patients [33]. However, according to Leiva-Gea et al. [33] the impaired gut permeability in T1D patients can be more attributed to the binding of Veillonella to colonic crypt cells rather than change in zonulin levels. Lactate produced by Veillonella is pushed to the luminal surface and weakens tight junctions [33].
4. Gut microbiota ingests and ferment fibers and produce short-chain fatty acids (SCFA) [50–52]. SCFAs enter the blood circulation and modulate T-reg differentiation; thus autoimmunity is prevented [5, 53–55].

With keeping these mechanisms in mind, now we are going to discuss the known attributable mechanism of some highlighted bacteria in more details:

Bacteroidetes, Firmicutes, Proteobacteria, and Actinobacteria were of great importance in our reviewed articles.

Genera Bacteroides and Prevotella are two important subgroups in Phylum Bacteroides that were increased in most of the T1D patient’s samples and can affect gut microbial composition by several mechanisms. Succinate and acetate are the main byproducts of anaerobic metabolism in this phylum that compromise epithelial tight junctions, decrease gut mucosal integrity, block T-reg differentiation and activates inflammatory pathways [25, 32, 33].

These bacteria also produce Glutamic acid decarboxylase (GAD) which can stimulate GAD autoimmunity by molecular mimicry [24, 32].

Phylum Actinobacteria including genus Bifidobacterium is butyrate-producing taxa that have anti-inflammatory effects and augments gut barrier by cytokine modulation [33]. This bacteria also induces T-reg development that results in immune response suppression by regulation of IL-10 production [18].

The third important phylum, Firmicutes, consists of eight notable subgroups: Veillonella, Roseburia, Ruminococcus, Lactobacillus, Blautia, Streptococcus, Faecalibacterium, and Staphylococcus.

Association of T1D and Veillonella is controversy. Kostic et al. [39] reported a decrease in Veillonella in T1D patients and proposed the following mechanism: reduced level of lithocholic acid results in stimulation of gut inflammation by an increased level of reactive oxygen species, reactive nitrogen species and nuclear factor-kB (NF-kB) activity in epithelial cells. Sphingomyelin increment also inhibits NK-T cell function that prevents inflammation [39].

Ruminococcaceae are butyrate-producing taxa that were reported declined in some studies and increased in others. The mechanism of reduced Ruminococcaceae in T1D patients is the same as Veillonella reduction mechanism [33, 39].

Faecalibacterium and Roseburia have anti-inflammatory effects, their presence may augment gut barrier function by modulating cytokine production and butyrate synthesis [24, 33, 36]. These genera have decreased in almost all patient samples.

Genus Blautia is also butyrate-producing taxa that have declined in most of the reviewed articles and plays an important role in blood glucose regulation, lipid metabolism and regulation of T-cell differentiation [18, 33]. There is also evidence of its increment in literature.

Genus Lactobacillus eliminates peroxidase radicals by superoxide dismutase and peroxidase enzymes thus provide a suitable condition for Bifidobacterium reproduction [28]. Lactobacillus down-modulate inflammation and previous studies have demonstrated that dendritic cells co-cultured with species of lactobacilli induce polarization of T-reg cells [5, 56, 57].

Staphylococcaceae may stimulate the growth of Bifidobacterium, Clostridium and Bacteroides which results in augmentation of neonate’s gut maturation [5, 58]. Streptococcaceae produce GAD so they have the same effects as Bacteroides [24, 59].

## Limitations

Limitations of our study that complicates interpretation of results can be listed as below: various geographical areas studied have an effect on diet of patients and controls; diversity of microbiota analysis techniques; colonizing microbiome and genetic susceptibility to T1D, some studies considered HLA as a genetic predisposing factor in the selection process of case and control individuals whereas others have ignored this point; different study design (e.g. some studies noticed seropositive group and seronegative FDRs in addition to T1D patients and healthy individuals while others just compared T1D patients with healthy individuals) [5, 25, 39, 44, 45]; various statistical analysis methods and different levels of p-value significance were reported in reviewed articles, however, in this study we used only statistical significant findings from the articles included.

## Suggestions

According to our study, we suggest new therapeutic and diagnostic strategies that need further clinical trials for assessing their effectiveness:

1. Use of prebiotics, probiotics and fecal microbial transplantation to modulate gut microbiome; e.g. a probiotic mixture of certain bacteria (mentioned in “Results”) can reduce HbA1c level so it can be considered as a complementary strategy for T1D management.
2. In order to detect early evidence of dysbiosis and prevention of T1D progression, serial stool exams in genetically susceptible children can be done by using a specific kit that semi-quantitatively compares microbiota composition of healthy control and suspected individual. In designing the kit, Firmicutes: Bacteroidetes ratio should be considered because it has been reported decreased in all the reviewed studies.

## Conclusions

Our study showed a significant association between alterations in intestinal microbial composition and T1D; however, in some articles, it is not clear which one happens first. Investigation of altered gut microbiota can help in the early detection of T1D before seropositivity against classical autoantigens. Targeted microbiome modulation can be a novel potential therapeutic strategy.

## Data Availability

All data were included.
